# Impaired surface marker expression in stimulated Epstein-Barr virus transformed lymphoblasts from Barth Syndrome patients

**DOI:** 10.1038/s41598-022-10270-4

**Published:** 2022-04-13

**Authors:** Hana M. Zegallai, Grant M. Hatch

**Affiliations:** 1 Department of Pharmacology and Therapeutics, University of Manitoba, Children’s Hospital Research Institute of Manitoba, Winnipeg, MB R3E3P4 Canada; 2 John Buhler Research Center, 501C-715 Bannatyne Avenue, Winnipeg, MB R3E3P4 Canada

**Keywords:** Biochemistry, Biological techniques, Cell biology, Immunology, Molecular biology, Medical research

## Abstract

Primary B lymphocytes rapidly respond to lipopolysaccharide (LPS) and cytosine linked to a guanine by a phosphate bond deoxyribonucleic acid (CpG DNA) stimulation to promote adaptive immune function through increased surface marker expression. Here we examined expression of surface markers in LPS and CpG DNA stimulated Epstein-Barr virus transformed B lymphoblasts from control and BTHS patients with different mutations. The percentage of cluster of differentiation (CD) positive cells including CD38 + , CD138 + , CD80 + surface expression and programmed cell death protein 1 (PD1 +) surface expression was similar between control and BTHS lymphoblasts incubated plus or minus LPS. The percentage of CD24 + , CD38 + and CD138 + cells was similar between control and BTHS lymphoblasts incubated plus or minus CpG DNA. CD27 + surface marker expression was reduced in both BTHS lymphoblasts and controls incubated with CpG DNA and PD1 + surface marker expression was higher in BTHS cells compared to controls but was unaltered by CpG DNA treatment. Thus, Epstein-Barr virus transformed control and BTHS lymphoblasts fail to increase selected surface markers upon stimulation with LPS and exhibit variable surface marker expression upon stimulation with CpG DNA. Since B lymphocyte surface marker expression upon activation is involved in B cell proliferation and differentiation, cell–cell interaction and the adaptive immune response, we suggest that caution should be exercised when interpreting immunological data obtained from Epstein-Barr virus transformed BTHS cells. Based upon our observations in control cells, our conclusions may be more broadly applicable to other diseases which utilize transformed B lymphocytes for the study of immune biology.

## Introduction

Barth Syndrome (BTHS) is a rare X-linked genetic disease caused by a mutation in the TAFAZZIN gene which codes for the cardiolipin (CL) transacylase protein tafazzin^1–3^. Tafazzin remodels nascent de novo synthesized CL into a form of CL found in the tissue specific mitochondrial membrane^4^. As the signature lipid of mitochondria, CL is required to support many mitochondrial functions including energy production required for adaptive immunity of B lymphocytes^5,6^. Although cardiomyopathy is the major cause of mortality in BTHS, many patients suffer from severe infections due to neutropenia^1,2^.

Several laboratories, including our own, have utilized Epstein-Barr virus transformed B lymphoblasts from patients for the study of BTHS pathology^7–11^. The rationale for the use of these cells is that they are easy to maintain in culture and the transformed nature of these cells makes them readily amenable to experimental manipulation. In addition, they are representative of the specific mutation of a given patient. However, studies have also indicated that Epstein-Barr virus transformation of human B lymphocytes may hinder host immune function^12^. B lymphoblasts express Toll-like receptors (TLRs) including TLR4 which can be stimulated with lipopolysaccharide (LPS)^13^. In addition, cytosine linked to a guanine by a phosphate bond deoxyribonucleic acid (CpG DNA) activates human B lymphocytes through TLR9 regardless of whether the DNA is in the form of genomic bacterial DNA or in the form of a synthetic oligodeoxynucleotide^14^. In this study, we examined whether Epstein-Barr virus transformed control and BTHS B lymphoblasts express surface markers indicative of activation by LPS and CpG DNA. We show that selected surface marker expression is refractory to stimulation with LPS and variable to stimulation with CpG DNA in Epstein-Barr virus transformed human B lymphoblasts from control and BTHS patients. Since B lymphocyte surface marker expression upon activation is required for B cell development, cell–cell interaction with other immune cells and the adaptive immune response, our results suggest that caution should be exercised when interpreting immunological data obtained from Epstein-Barr virus transformed B lymphoblasts from BTHS patients.

## Materials and methods

Epstein-Barr virus transformed human control lymphoblasts and Epstein-Barr virus transformed BTHS lymphoblasts were obtained from the Coriell Institute for Medical Research (Camden, NJ, USA). The cells that were used in the study are outlined in Table 1. RPMI 1640 media, Fetal Bovine Serum (FBS), 1% Antibiotic–Antimycotic (A/A), and Propidium Iodide (PI) were obtained from Life Technologies Inc. (Burlington, ON, Canada). Anti-CD19-APC, anti-CD24-BV421, anti-CD27-perCP-CY5.5, anti-CD38-APC-H7, anti-CD138-PE, anti-CD80 PE-A, and anti-PD1-APC antibodies for flow cytometry analysis were purchased from BD Biosciences (Seattle, WA, USA). Unless otherwise indicated, all other reagents used were of analytical grade and were obtained from either Thermo Fisher Scientific (Winnipeg, MB) or Sigma-Aldrich (Oakville, ON).

**Table 1:** Cell lines used in this study.

Cell line (Identifier)	Phenotype	TAFAZZIN mutation	Age at harvest
07,535	Healthy control	None	15 years
07,491	Healthy control	None	17 years
16,408	Healthy control	None	12 years
22,192	BTHS	Exon 3, Substitution of cystine for arginine	10 years
22,193	BTHS	Exon 6, premature stop codon	10 years
22,194	BTHS	Complete deletion of TAFAZZIN gene	9 years

### Cell culture and stimulation

All work was performed with approval from the University of Manitoba Environmental Health and Safety Office (Biological Safety Project Approval Certificate #BB0044-2). Cells were grown in RPMI-1640 medium supplemented with 15% FBS and 1% A/A at 5% CO_2_ at 37 °C in a Thermo Scientific CO_2_ incubator HEPA Class 200. The medium was replaced every 48 h and the cells were passaged every five days. Cells were pelleted by centrifugation at 1400 rpm for 10 min at room temperature. Cells were then washed twice with phosphate buffered saline (PBS) prior to experimental stimulation. Control and BTHS Lymphoblasts were incubated plus or minus 10 µg/ml lipopolysaccharide (LPS) (E. Coli 128: B12) for 24 h or 5 µM CpG DNA (ODN 2006) (TLR9 selective) for 24 h. After stimulation, the cells were harvested by centrifugation as above and washed twice with PBS prior to further analysis.

### Cell viability and surface marker expression analysis

Briefly, after 24 h of stimulation with LPS or CpG DNA, the lymphoblasts were centrifuged at 1400 rpm for 10 min. The pellets were washed with PBS and suspended in 100 µl of PBS. Lymphoblasts were stained with propidium iodide (5 µg/ml) for 5 min in the dark at 4 °C. This was followed by flow cytometry analysis. Surface marker expression was measured in untreated and stimulated lymphoblasts by staining the cell surface with anti-CD19-APC, anti-CD24-BV421, anti-CD27-perCP-CY5.5, anti-CD38-APC-H7, anti-CD138-PE, anti-CD80 PE-A, and anti-PD1-APC as per the manufacturer’s instructions. Cells were then analyzed by flow cytometry at the Flow Cytometry Core Facility in the Rady Faculty of Health Sciences, University of Manitoba, using a BD FACS Canto II instrument. FlowJo software was used for data analysis.

### Statistical analysis

All data are expressed as mean ± SD. Comparison between two groups was performed by using Two tailed unpaired Student’s t test, and comparison between multiple groups was achieved using one-way analysis of variance (ANOVA) followed by Tukey’s post-hoc multiple comparison test. A *p* value of < 0.05 was considered statistically significant.

## Results

### Cell viability is maintained in Epstein-Barr virus transformed human control and BTHS B lymphoblasts after stimulation

**Figure 1:**
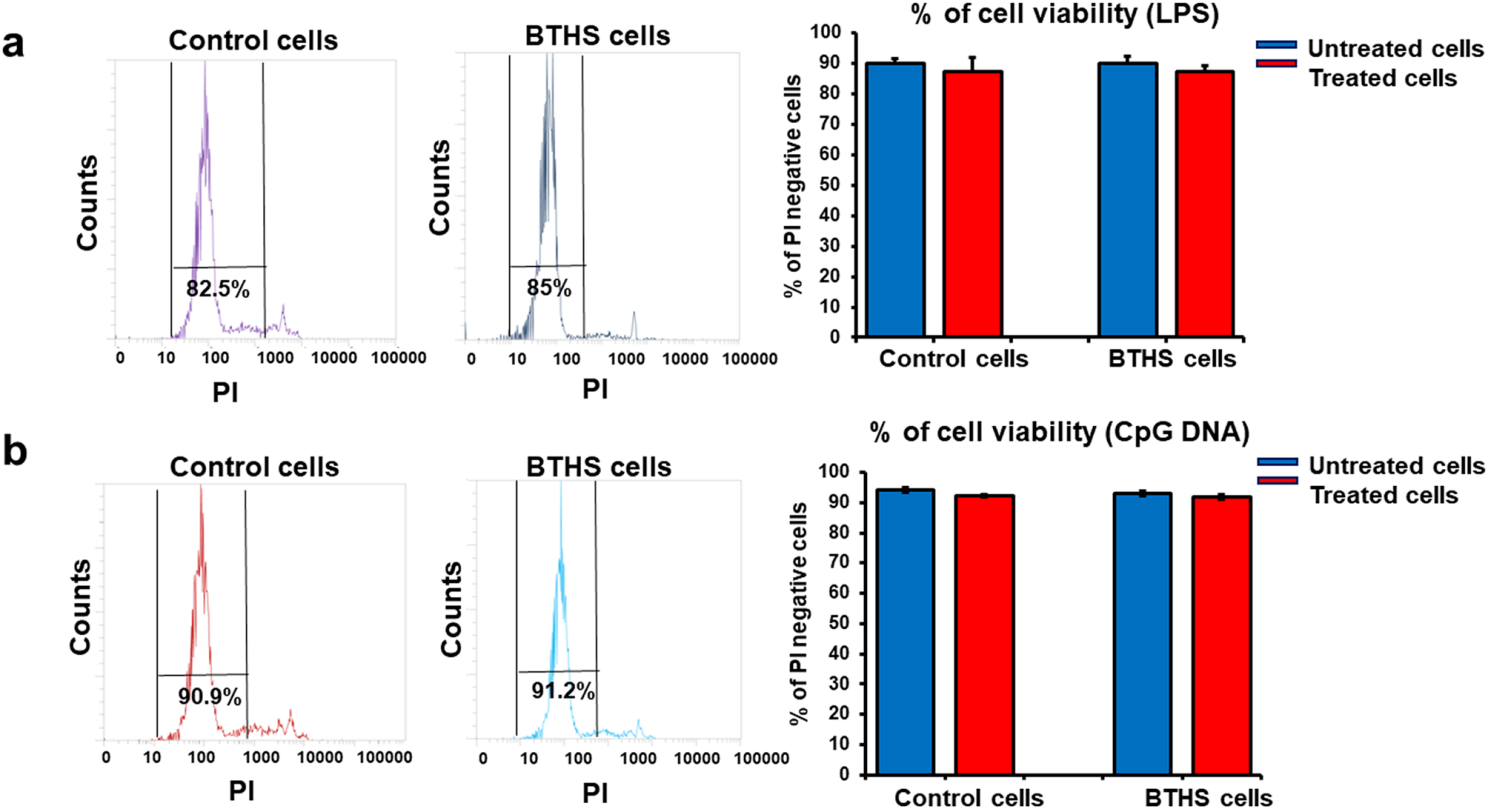
Cell viability is maintained in both Epstein-Barr virus transformed human control and BTHS lymphoblasts after stimulation. Cell viability was measured in control and BTHS lymphoblasts after stimulation with 10 µg/ml LPS or 5 µM CpG DNA using flow cytometry as described in “Materials and Methods”. (**a**) Representative flow cytometry images of the percentage of cell viability in untreated and LPS treated cells. (**b**) Representative flow cytometry images of the percentage of cell viability in untreated and CpG DNA treated cells. *N* = 3.

Initially we examined viability of LPS and CpG DNA treated control and BTHS B lymphoblasts. Epstein-Barr virus transformed B lymphoblasts from 3 control patients and 3 BTHS patients with different mutations were incubated for 24 h with 10 µg/ml LPS or 5 µM CpG DNA then stained with propidium iodide and flow cytometry performed to assess cell viability. We observed that cell viability was maintained in both control and BTHS lymphoblasts incubated with 10 µg/ml LPS (Fig. 1a) or 5 µM CpG DNA (Fig. 1b) for 24 h. Thus, cell viability is maintained in Epstein-Barr virus transformed human control and BTHS B lymphoblasts after stimulation with LPS or CpG DNA.

### Epstein-Barr virus transformed human control and BTHS B lymphoblasts surface marker expression is refractory to stimulation with LPS

**Figure 2:**
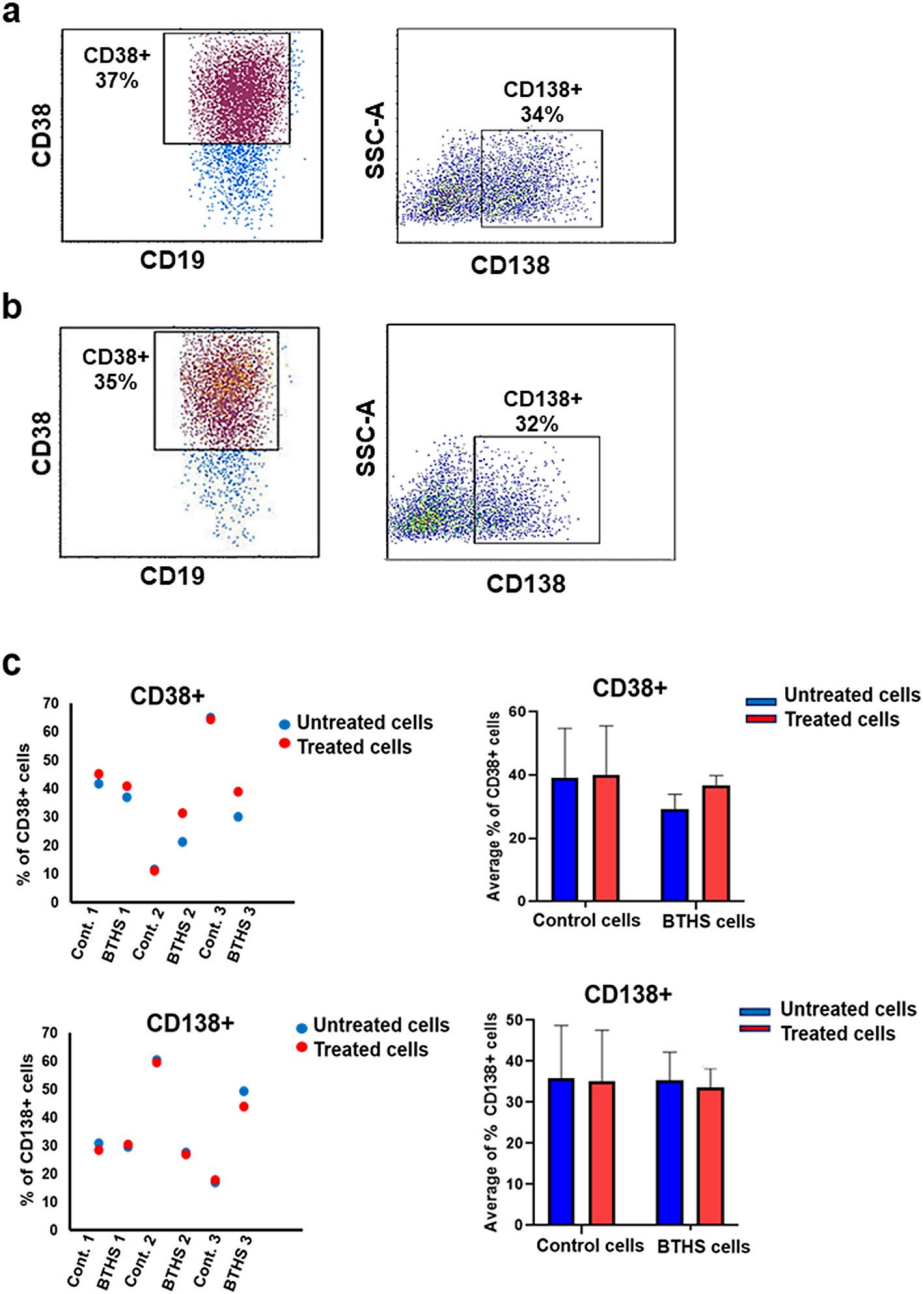
CD38 + and CD138 + surface markers expression in Epstein-Barr virus transformed human age-matched control and BTHS lymphoblasts after LPS stimulation. The expression of CD38 + and CD138 + surface markers were determined in control and BTHS lymphoblasts after stimulation without or with 10 µg/ml LPS using flow cytometry as described in “Materials and Methods”. (**a**) Representative flow cytometry images of CD38 + and CD138 + surface markers in control cells after LPS stimulation. (**b**) Representative flow cytometry images of CD38 + and CD138 + surface markers in BTHS cells after LPS stimulation. (**c**) Histograms showing percentage of CD38 + and CD138 + surface markers expression in LPS treated control and BTHS cells compared to untreated control and BTHS cells. *N* = 3.

**Figure 3:**
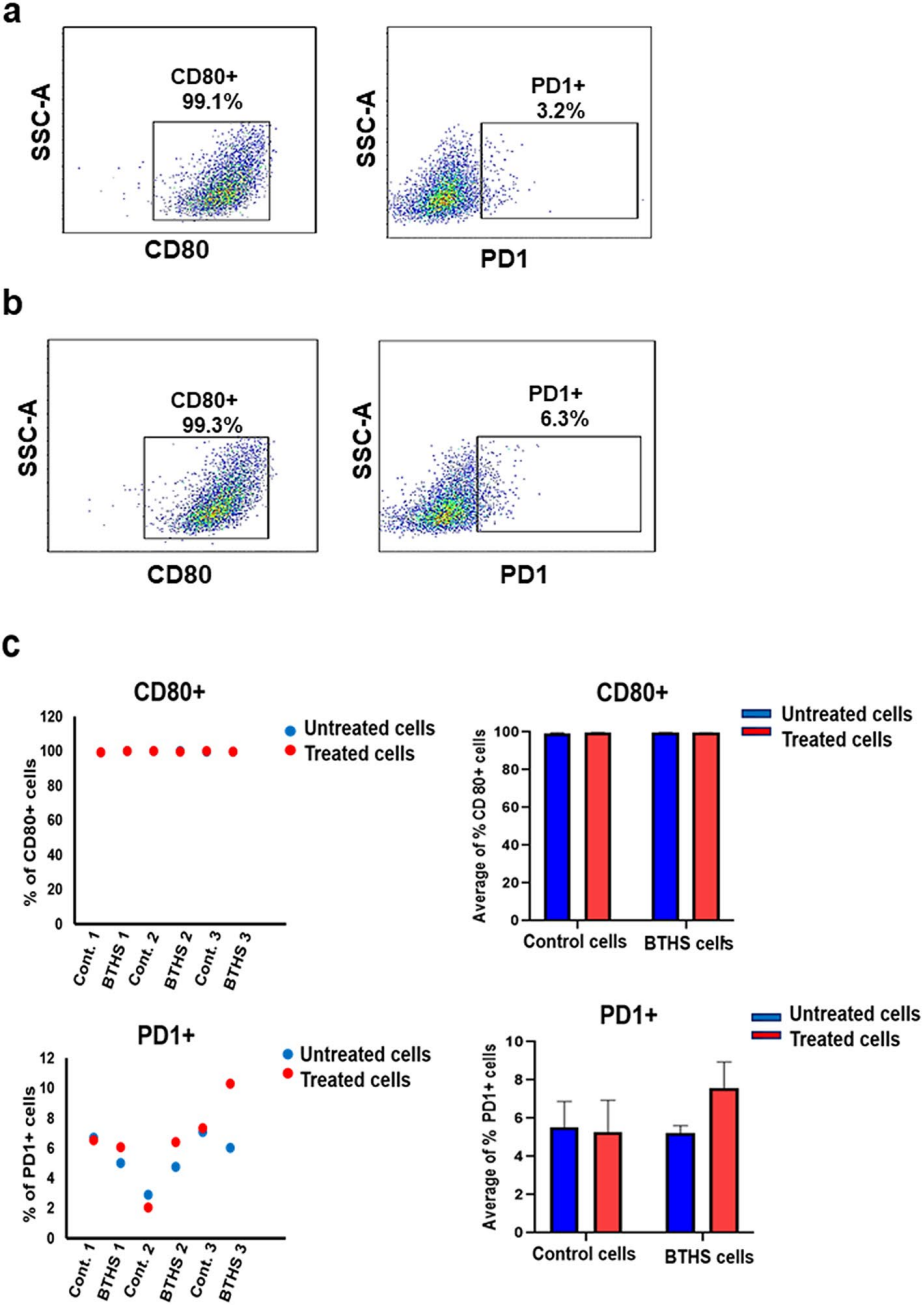
CD80 + and PD1 + surface markers expression in Epstein-Barr virus transformed human age-matched control and BTHS lymphoblasts after LPS stimulation. The expression of CD80 + and PD1 + surface markers were determined in control and BTHS lymphoblasts after stimulation without or with 10 µg/ml LPS using flow cytometry as described in “Materials and Methods”. (**a**) Representative flow cytometry images of CD80 + and PD1 + surface markers in control cells after LPS stimulation. (**b**) Representative flow cytometry images of CD80 + and PD1 + surface markers in BTHS cells after LPS stimulation. (**c**) Histograms showing percentage of CD80 + and PD1 + surface markers expression in LPS treated control and BTHS cells compared to untreated control and BTHS cells. *N* = 3.

Control and BTHS B lymphoblasts were incubated minus or plus 10 µg/ml LPS for 24 h and expression of the surface markers CD38 + , CD138 + , CD80 + and PD1 + determined. The percentage of CD38 + and CD138 + surface expression was similar between control and BTHS B lymphoblasts incubated minus or plus LPS for 24 h (Fig. 2a–c). In addition, the percentage of CD80 + and PD1 + surface expression was similar between control and BTHS B lymphoblasts incubated minus or plus LPS for 24 h (Fig. 3a–c). Thus, selected surface marker expression in Epstein-Barr virus transformed human control and BTHS B lymphoblasts was refractory to stimulation with LPS.

### Epstein-Barr virus transformed human control and BTHS B lymphoblasts show variable surface marker expression upon CpG DNA stimulation

**Figure 4:**
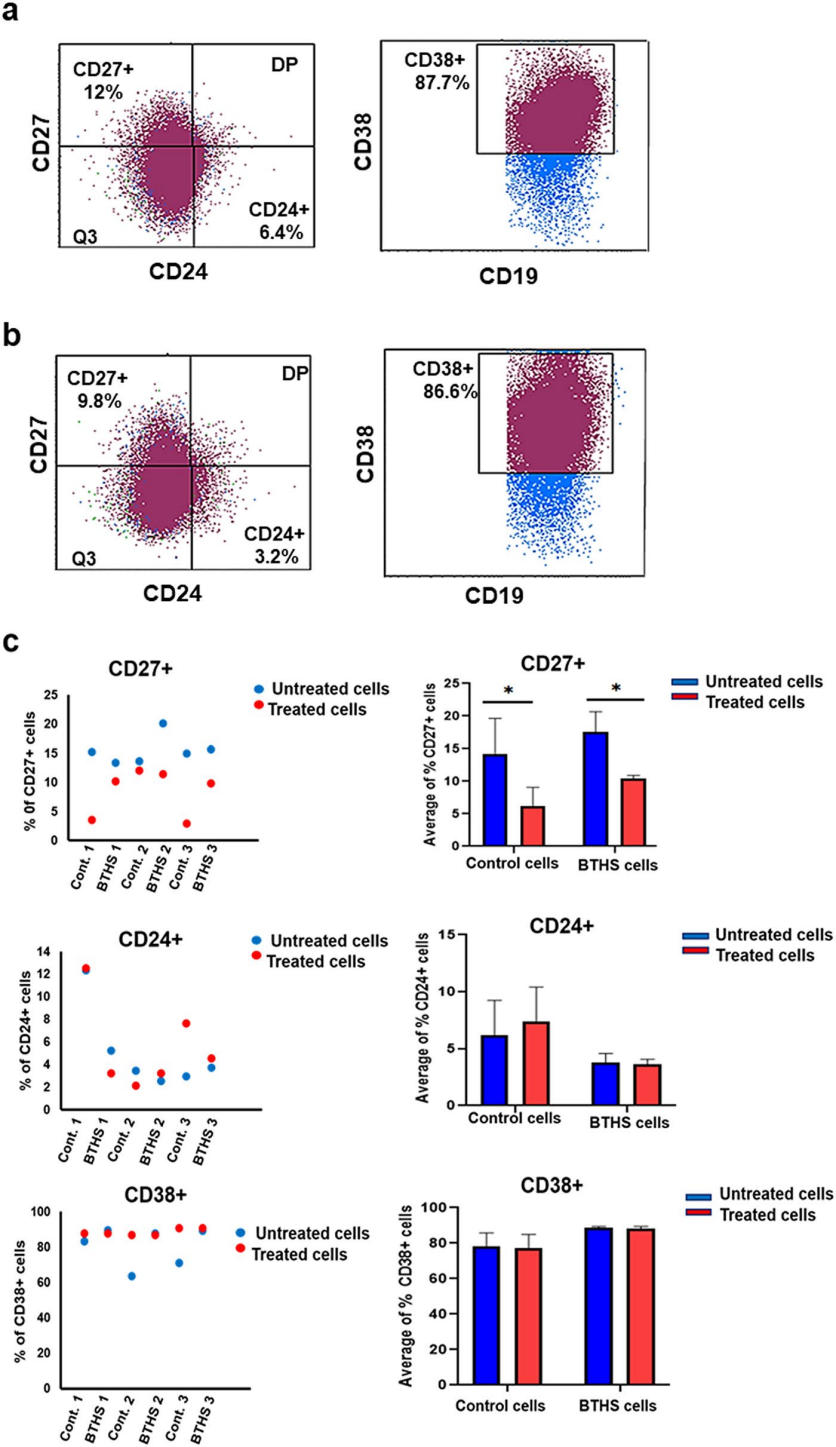
Epstein-Barr virus transformed human age-matched control and BTHS B lymphoblasts show variable expression of CD27 + , CD24 + , and CD38 + surface markers upon CpG DNA stimulation. The expression of CD27 + , CD24 + , and CD38 + surface markers was determined in control and BTHS cells after stimulation without or with 5 µM CpG DNA using flow cytometry as described in “Materials and Methods”. (**a**) Representative flow cytometry images of CD27 + , CD24 + , and CD38 + surface markers in control cells after CpG DNA stimulation. (**b**) Representative flow cytometry images of CD27 + , CD24 + , and CD38 + surface markers in BTHS cells after CpG DNA stimulation. (**c**) Histograms showing percentage of CD27 + , CD24 + , and CD38 + surface marker expression in CpG DNA treated control and BTHS cells compared to untreated control and BTHS cells. *N* = 3, *p < 0.05.

**Figure 5:**
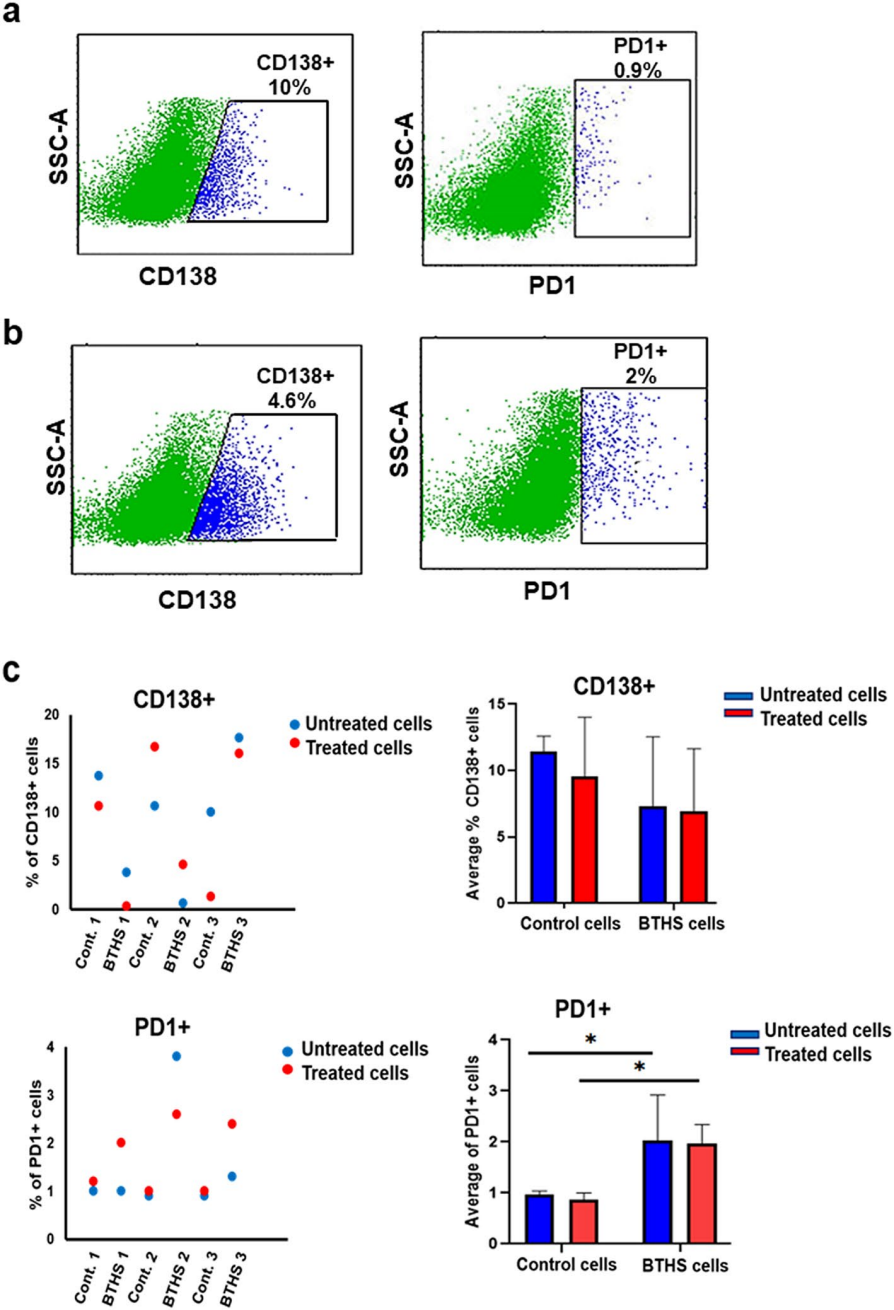
CD138 + and PD1 + surface markers expression in Epstein-Barr virus transformed human age-matched control and BTHS lymphoblasts after CpG DNA stimulation. The expression of CD138 + and PD1 + surface markers were determined in control and BTHS cells after stimulation without or with 5 µM CpG DNA using flow cytometry as described in “Materials and Methods”. (**a**) Representative flow cytometry images of CD138 + and PD1 + surface markers in control cells after CpG DNA stimulation. (**b**) Representative flow cytometry images of CD138 + and PD1 + surface markers in BTHS cells after CpG DNA stimulation. (**c**) Histograms showing percentage of CD138 + and PD1 + surface marker expression in CpG DNA treated control and BTHS cells compared to untreated control and BTHS cells. *N* = 3, *p < 0.05.

Control and BTHS B lymphoblasts were incubated minus or plus 5 µM CpG DNA for 24 h and expression of the surface markers CD24 + , CD38 + , CD138 + , CD27 + and PD1 + determined. The percentage of CD24 + and CD38 + was similar between control and BTHS B lymphoblasts incubated minus or plus 5 µM CpG DNA for 24 h (Fig. 4a–c). In contrast, CD27 + surface marker expression was reduced in both control and BTHS B lymphoblasts incubated with 5 µM CpG DNA for 24 h. The percentage of CD138 + expression was similar between control and BTHS B lymphoblasts incubated minus or plus 5 µM CpG DNA for 24 h (Fig. 5a–c). In contrast, the percentage of PD1 + expression was higher in BTHS cells compared to controls but was unaltered by incubation with 5 µM CpG DNA for 24 h. Thus, Epstein-Barr virus transformed human control and BTHS B lymphoblasts showed variable selected surface marker expression upon CpG DNA stimulation.

## Discussion

In this study we examined whether Epstein-Barr virus transformed control and BTHS B lymphoblasts expressed surface markers indicative of classical B lymphocyte activation upon LPS or CpG DNA stimulation. Surprisingly, our results clearly demonstrate that Epstein-Barr virus transformed human control and BTHS B lymphoblasts are refractory to selected surface marker expression upon stimulation with LPS and exhibit variable selected surface marker expression upon stimulation in response to CpG DNA.

CD24 is expressed at the surface of activated B lymphocytes and is required for B cell development and plays a key role in cell–cell adhesion^15^. In addition, CD24 expression on B cells is required for T cell co-stimulation in CD4 T-cell mediated clonal expansion^16^. CD38 is a key surface marker for B cell activation and plays an important role in B cell proliferation and regulation^17^. Previous studies showed that deficiency of CD38 expression resulted in inhibition of the immune response and increased susceptibility to infections^18^. CD138 is a specific surface marker of plasma cells which are required for long-term humoral immunity^19^. Upon stimulation, B cells differentiate into antibody secreting cells and upregulate CD138 on their surface which enhances antibody secreting cell maturation and accumulation^20^. CD80 is expressed in activated B cells and provides a co-stimulatory signal necessary for T cell activation^21^. A previous global gene expression profile analysis demonstrated significant differences in the gene expression patterns between Epstein-Barr virus transformed and normal host lymphocytes and that transformation may hinder normal immune function^12^. We recently demonstrated that isolated naïve primary murine B lymphocytes stimulated with LPS readily increased surface marker expression and that tafazzin deficiency in these cells markedly impaired surface marker expression upon stimulation with LPS^22^. The lack of elevated surface marker expression in human control and BTHS lymphoblasts in response to LPS, coupled with the fact that these surface markers were readily detectable in unstimulated cells, indicate that these cells exhibit impaired responsiveness to LPS-mediated activation.

CD27 is a B lymphocyte memory marker and a co-stimulatory immune molecule which belongs to the tumor necrosis factor receptor superfamily^23^. CD27 surface marker expression plays an important role in immunoglobulin synthesis and in regulating B cell activation through binding with CD70 on activated B and T lymphocytes and modulates induction of apoptosis^24,25^. PD1 surface marker expression down regulates the immune system to limit inflammation by modulating T-cell activity^26^. For example, PD1 expressed on the surface of activated B cells inhibited CD4 + and CD8 + T cell proliferation^27^. In addition, inhibition of the PD1 pathway resulted in increased B cell activation, proliferation and secretion of inflammatory cytokines^28^. Intriguingly, CD27 surface expression was reduced in both control and BTHS B lymphoblasts stimulated with CpG DNA. In contrast, PD1 surface expression was higher in BTHS cells compared to controls but was unaltered by stimulation with CpG DNA. These data suggest that Epstein-Barr virus transformed human control and BTHS B lymphoblasts exhibit variability in surface marker expression upon CpG DNA stimulation.

The significance of our observations is currently unknown. However, Epstein-Barr virus infection of human B lymphocytes was recently shown to promote major modifications of alternative splice variant expression which may impact on cell fate determination^29^. In addition, the differences in gene expression patterns observed between Epstein-Barr virus transformed and normal lymphocytes^12,30^, coupled with our observation of impaired B cell surface marker expression, could potentially influence transformed B lymphocyte function when infection is mimicked through LPS- or CpG DNA-mediated activation. Thus, the Epstein-Barr virus transformed BTHS lymphoblast phenotype may exhibit a lack of responsiveness to pro-inflammatory stimuli and potentially attenuate activation of other immune cells. For example, activated B lymphocytes secret chemokines which recruit neutrophils to sites of infection and Epstein-Barr virus infection downregulates the expression of CXCL1^12^. Hence, impaired B cell activation in Epstein-Barr virus transformed BTHS lymphoblasts could potentially attenuate neutrophil recruitment. Another conclusion from our study, derived from the behavior of the controls, is that it can be assumed that the effects are not necessarily limited to BTHS but are more broadly applicable to other diseases which utilize Epstein-Barr virus transformed B lymphocytes for the study of immune pathology. In summary, based upon our observations we suggest that caution should be exercised when interpreting immunological data obtained from Epstein-Barr virus transformed lymphoblasts.

## References

[1] Clarke SL (2013). Barth syndrome. Orphan. J. Rare Dis..

[2] Adès LC (1993). Barth syndrome: Clinical features and confirmation of gene localisation to distal Xq28. Am. J. Med. Genet..

[3] Bione S (1996). A novel X-linked gene, G4.5. is responsible for Barth syndrome. Nat. Genet..

[4] Xu Y, Malhotra A, Ren M, Schlame M (2006). The enzymatic function of Tafazzin. J. Biol. Chem..

[5] Zegallai HM, Hatch GM (2021). Barth syndrome: cardiolipin, cellular pathophysiology, management, and novel therapeutic targets. Mol. Cell. Biochem..

[6] Sandoval H, Kodali S, Wang J (2018). Regulation of B cell fate, survival, and function by mitochondria and autophagy. Mitochond..

[7] Mejia EM (2018). Expression of human monolysocardiolipin acyltransferase-1 improves mitochondrial function in Barth syndrome lymphoblasts. J. Biol. Chem..

[8] Hauff KD, Hatch GM (2010). Reduction in cholesterol synthesis in response to serum starvation in lymphoblasts of a patient with Barth syndrome. Biochem. Cell Biol..

[9] Gonzalvez F (2013). Barth syndrome: Cellular compensation of mitochondrial dysfunction and apoptosis inhibition due to changes in cardiolipin remodeling linked to tafazzin (TAZ) gene mutation. Biochim. Biophys. Acta (BBA) - Mol Basis Dis..

[10] Saric A, Andreau K, Armand A-S, Møller IM, Petit PX (2016). Barth syndrome: From mitochondrial dysfunctions associated with aberrant production of reactive oxygen species to pluripotent stem cell studies. Front. Genet..

[11] Yu Y (2005). Characterization of lymphoblast mitochondria from patients with Barth syndrome. Lab. Invest..

[12] Dai Y (2012). Screening and functional analysis of differentially expressed genes in EBV-transformed lymphoblasts. Virol. J..

[13] Siennicka J, Trzcinska A, Czescik A, Dunal-Szczepaniak M, Łagosz B (2013). The influence of toll-like receptor stimulation on expression of EBV lytic genes. Pol. J. Microbiol..

[14] Krieg AM (2002). CpG Motifs in bacterial DNA and their immune effects. Annu. Rev. Immunol..

[15] Baumann P (2005). CD24 expression causes the acquisition of multiple cellular properties associated with tumor growth and metastasis. Cancer Res..

[16] Fang X, Zheng P, Tang J, Liu Y (2010). CD24: From A to Z. Cell. Mol. Immunol..

[17] Malavasi F (2008). Evolution and function of the ADP Ribosyl Cyclase/CD38 gene family in physiology and pathology. Physiol. Rev..

[18] Glaría E, Valledor AF (2020). Roles of CD38 in the immune response to infection. Cells.

[19] Pioli PD (2019). Plasma cells, the next generation: Beyond antibody secretion. Front. Immunol..

[20] McCarron MJ, Park PW, Fooksman DR (2017). CD138 mediates selection of mature plasma cells by regulating their survival. Blood.

[21] Good-Jacobson KL, Song E, Anderson S, Sharpe AH, Shlomchik MJ (2012). CD80 expression on B cells regulates murine T follicular helper development, germinal center B cell survival, and plasma cell generation. J. Immunol..

[22] Zegallai HM (2021). Tafazzin deficiency impairs mitochondrial metabolism and function of lipopolysaccharide activated B lymphocytes in mice. FASEB J..

[23] Agematsu K, Hokibara S, Nagumo H, Komiyama A (2000). CD27: A memory B-cell marker. Immunol. Today.

[24] Kobata T, Jacquot S, Kozlowski S, Agematsu K, Schlossman SF, Morimoto C (1995). CD27-CD70 interactions regulate B-cell activation by T cells. Proc. Natl. Acad. Sci. USA.

[25] Prasad KV (1997). CD27, a member of the tumor necrosis factor receptor family, induces apoptosis and binds to Siva, a proapoptotic protein. Proc. Natl. Acad. Sci. USA.

[26] Trivedi MS, Hoffner B, Winkelmann JL, Abbott ME, Hamid O, Carvajal RD (2015). Programmed death 1 immune checkpoint inhibitors. Clin. Adv. Hematol. & Oncol..

[27] Wang X (2019). PD-1-expressing B cells suppress CD4+ and CD8+ T cells via PD-1/PD-L1-dependent pathway. Mol. Immunol..

[28] Thibult M-L (2013). PD-1 is a novel regulator of human B-cell activation. Intl. Immunol..

[29] Manet E (2021). Modulation of alternative splicing during early infection of human primary B lymphocytes with Epstein-Barr virus (EBV): a novel function for the viral EBNA-LP protein. Nuc. Acids Res..

[30] Tang Y (2019). Bioinformatic analysis of differentially expressed genes and identification of key genes in EBV-transformed lymphoblasts. Biomed. Pharmacother..

